# Nudivirus Sequences Identified from the Southern and Western Corn Rootworms (Coleoptera: Chrysomelidae)

**DOI:** 10.3390/v13020269

**Published:** 2021-02-09

**Authors:** Sijun Liu, Thomas W. Sappington, Brad S. Coates, Bryony C. Bonning

**Affiliations:** 1 Department of Entomology, Iowa State University, Ames, IA 50011, USA; sliu@iastate.edu; 2 Corn Insects and Crop Genetics Research Unit, USDA-ARS, Ames, IA 50011, USA; tom.sappington@usda.gov (T.W.S.); brad.coates@usda.gov (B.S.C.); 3 Department of Entomology and Nematology, University of Florida, Gainesville, FL 32611, USA

**Keywords:** *Nudiviridae*, *Alphanudivirus*, beetle, corn rootworm, *Diabrotica* spp.

## Abstract

Analysis of pooled genomic short read sequence data revealed the presence of nudivirus-derived sequences from U.S. populations of both southern corn rootworm (SCR, *Diabrotica undecimpunctata howardi* Barber) and western corn rootworm (WCR, *Diabrotica virgifera virgifera* LeConte). A near complete nudivirus genome sequence was assembled from sequence data for an SCR population with relatively high viral titers. A total of 147,179 bp was assembled from five contigs that collectively encode 109 putative open reading frames (ORFs) including 20 nudivirus core genes. In contrast, genome sequence recovery was incomplete for a second nudivirus from WCR, although sequences derived from this virus were present in three geographically dispersed populations. Only 48,989 bp were assembled with 48 putative ORFs including 13 core genes, representing about 20% of a typical nudivirus genome. Phylogenetic analysis indicated that both corn rootworm nudiviruses grouped with the third known nudivirus of beetles, *Oryctes rhinoceros nudivirus* in the genus *Alphanudivirus*. On the basis of phylogenetic and additional analyses, we propose further taxonomic separation of nudiviruses within *Alphanudivirus* and *Betanudivirus* into two subfamilies and five genera. Identification of nudivirus-derived sequences from two species of corn rootworm highlights the diversity of viruses associated with these agricultural insect pests.

## 1. Introduction

Corn rootworms are major insect pests of corn in the Americas and Europe [1]. The four North American corn rootworm species and subspecies are the western corn rootworm (WCR, *Diabrotica virgifera virgifera* LeConte), Mexican corn rootworm (MCR, *Diabrotica virgifera zeae* Krysan & Smith), and northern corn rootworm (NCR, *Diabrotica barberi* Smith & Lawrence) in the *virgifera* species group of *Diabrotica* beetles; and the southern corn rootworm (SCR, *Diabrotica undecimpunctata howardi* Barber) in the *fucata* species group [2]. Of these, WCR, MCR, and NCR can cause devastating damage to corn, and are serious threats to agricultural productivity in the U.S. Together, these pests cause losses estimated at nearly $2 billion annually in revenue in the U.S. (75% in yield loss, 25% in treatment costs) [3]. Traditionally, WCR, MCR, and NCR have been managed through crop rotation, extensive application of insecticides to the soil to kill the underground larvae, and, in some regions, late-season aerial applications of insecticides targeting egg-laying adults. However, biotypes have evolved that are adapted to crop rotation, and in areas of high insecticide use, insecticide resistance has become a problem [4]. More recently, transgenic corn varieties expressing WCR-active pesticidal proteins derived from the bacterium *Bacillus thuringiensis* (Bt) have been widely adopted, but resistant populations have evolved in Iowa and Nebraska within the U.S. [5,6,7]. New transgenic lines have been developed that use a combination of gut-active Bt-derived proteins and silencing RNAs to silence essential rootworm genes through RNA interference (RNAi) [8,9,10]. While promising, there are indications that WCR will also readily develop resistance and cross-resistance to RNAi corn varieties, and care must be taken to preserve their efficacy after deployment [11,12]. Given the significant losses resulting from rootworm damage, and limitations associated with current management techniques, new tools to suppress rootworm populations are urgently needed. Pathogenic viruses could play a role in rootworm management as demonstrated for another coleopteran pest, Asiatic rhinoceros beetle, *Oryctes rhinoceros* (L.) [13,14].

Little is known about the DNA viruses that infect *Diabrotica* spp. Virus particles have been observed in the spermatheca of WCR [15], and baculovirus-like and filamentous virus-like particles have been described from hemocytes and from the midgut, respectively, of SCR [16]. As no occlusion bodies typical of nucleopolyhedroviruses or granuloviruses were observed, the baculovirus-like particles observed in SCR were likely derived from a nudivirus. No pathologic symptoms were observed in beetles infected by these viruses [17,18].

*Nudiviridae*, formerly known as “non-occluded” baculoviruses, is a family of large, rod-shaped viruses with genomes composed of circular, double-stranded DNA (dsDNA). Nudivirus genomes range from 97 to 232 kbp and encode 93–154 putative proteins [19]. Nudiviruses are classified into two genera, *Alphanudivirus* and *Betanudivirus.* Members of *Alphanudivirus* have been isolated from insects, while the betanudiviruses infect both insects and marine arthropods (Amphipods and Decapods). Nudivirus-like viral particles have been widely observed, including from 10 insect orders (Coleoptera, Lepidoptera, Orthoptera, Diptera, Siphonaptera, Hymenoptera, Thysanura, Trichoptera, Neuroptera, and Hemiptera), and three additional arthropod orders (Acarina, Araneina, and Crustacea) [20]. A total of 13 nudiviruses have been sequenced (Table 1). The *Oryctes rhinoceros nudivirus* (OrNV), isolated from *O. rhinoceros*, is the only fully sequenced and annotated nudivirus from a coleopteran host. Short sequences derived from a second putative beetle-infecting nudivirus were isolated from the Japanese rhinoceros beetle, *Allomyrina dichotoma* (Table 1).

In contrast to the exogenous nudiviruses described above, nudiviral genome sequence fragments are also frequently integrated into the genome of the host insect [21]. Such an association gave rise to the *Bracovirus* genus of *Polydnaviridae* with sequences integrated into the genomes of parasitic wasps playing a critical role in the survival of wasp larvae within the parasitized host [22,23,24,25]. Such endogenous nudiviruses have also been documented in several hemipteran insects [26,27].

Our goal was to characterize the virome of two corn rootworm species, drawing on genomic and transcriptomic sequence data [42] toward the identification of viruses with potential for use in rootworm management. From this work, sequences derived from three novel small RNA viruses were identified from the WCR transcriptome [43,44,45], and two from the SCR transcriptome [46,47]. Here, we report on sequences derived from two novel nudiviruses of SCR and WCR assembled from corresponding short genomic sequencing read data. Both viruses appear to be closely related to the third known coleopteran nudivirus, OrNV in the genus *Alphanudivirus.* Based on phylogenetic analysis and assessment of protein homology among nudiviruses, a revised taxonomic classification is proposed. This work expands upon the described diversity of viruses infecting corn rootworm species, with potential for use in management of these devastating pests.

## 2. Materials and Methods

### 2.1. Insect Collection, DNA Isolation, Library Preparation and Illumina Sequencing

Adult SCR were collected live from a field near Ames, IA, USA in late July 2012, transported to the laboratory where all samples were flash frozen in liquid nitrogen and stored at −80 °C. A total of 35 males and 36 females (*n* = 71) were pooled and ground to a powder in liquid nitrogen. DNA was extracted from 3.0 mg of the ground SCR sample using Qiagen DNeasy Blood and Tissue Extraction kits (Germantown, MD, USA), with modifications to avoid DNA shearing as described previously [48]. Purified DNA was submitted to the Iowa State University DNA Facility (Ames, IA, USA), where genomic DNA was size selected and used to generate ~500-bp insert libraries using the Illumina TruSeq v2 Library Construction Kit (Illumina, San Diego, CA, USA). Single-end 100-bp Illumina HiSeq2500 reads were generated. Data were received in raw fastq format and submitted to the National Center for Biotechnology Information (NCBI) Short Read Archive (BioProject PRJNA689866; SRA Accession SRR13364002) [48].

Three sets of WCR genomic sequencing data were downloaded from the NCBI SRA database (BioProject PRJNA222656). Each adult WCR sequence dataset represented one of three locations, and each sample from a location comprised 5 pooled individuals (Table 2). Sequences were determined by Flagel et al. by Illumina HiSeq 2000 (200-nt sequence reads) [49].

### 2.2. Sequence Assembly

The quality of the Illumina sequencing reads from SCR was assessed by FastQC (http://www.bioinformatics.babraham.ac.uk/projects/fastqc/), and raw reads were trimmed to remove adapter and low-quality nucleotides, as described previously [48]. The low-quality nucleotides (*q* < 30) were trimmed from raw WCR sequence reads using Trimmomatic (v0.36) [50]. Trinity (v2.6.6) [51] was used to separately assemble trimmed reads de novo from SCR and WCR libraries. Redundancy was reduced among the resulting contigs using the CAP3 assembler [52]. Nudiviral-associated contigs were manually analyzed and further joined based on sequence overlap. To estimate read coverage of nudiviral sequences, trimmed sequence reads were mapped to the assembled nudiviral fragments with a Perl mapping script [26].

### 2.3. Identification of Contigs and Viral Sequence Discovery

Methods for annotation of the assembled DNA contigs for identification of virus-derived DNA sequences were as described previously [26]. Briefly, to identify potential viral sequences, the assembled contigs were queried against a local viral sequence database (built using the viral protein sequences extracted from the NCBI nr database) and filtered for BLASTx alignments with minimum *E*-value ≤ 0.001). Contigs with “hits” to viral proteins were extracted and used as BLASTx queries against the NCBI nr database. Results from BLASTx were analyzed to identify sequences derived from potential viruses. Based on the protein query results, nudiviral-derived sequences were either reassembled with the CAP3 DNA assembly program [52], or manually assembled by sequence alignment with BioEdit v7.2 [53] (https://bioedit.software.informer.com/). The nudiviral DNA sequences were further used to query the NCBI non-redundant nucleotide (nr/nt) database using the BLASTn algorithm, and alignments filter for *E*-values ≤ 0.001.

### 2.4. Viral Sequence Analysis and Annotation

Structural annotation of contigs derived from nudivirus sequences was performed via putative nudivirus open reading frame (ORF) translation using SnapGene Viewer (v4.1.9) (https://www.snapgene.com/). ORFs ≥ 50 aa with limited overlap were considered to be nudivirus-encoded genes as applied previously [33]. The selected ORFs were annotated using multiple BLAST algorithms including BLASTp, PSI-BLAST (Position-Specific Iterated BLAST), PHI-BLAST (Pattern Hit Initiated BLAST) or DELTA-BLAST (Domain Enhanced Lookup Time Accelerated BLAST) against the NCBI nr database. Viral genes encoding hypothetical proteins with unknown function were also analyzed by searching for conserved protein domains using the Conserved Domain Architecture Retrieval Tool (CDART) [54]. To assess interspecific similarities using BioEdit [53], translated proteins from SCR and WCR nudiviruses were used as BLASTp queries against a local BLAST database of nudivirus (proteins downloaded from NCBI and results filtered for *E*-values < 0.0001. Baculoviral core genes and nudivirus essential genes were used to construct a supermatrix phylogenetic tree as described previously [55]. The tree builder programs of the ETE Toolkit v3.0 [56] were used with default parameters employing the JTT (Jones–Taylor–Thorton) or WAG (Whelan and Goldman) models for maximizing the tree’s likelihood, and the resulting phylogeny viewed using FigTree (v1.4.4) (http://tree.bio.ed.ac.uk/software/figtree/).

## 3. Results

### 3.1. Assembly of Genomic Sequence Data for Nudiviral Sequence Identification

The genome sequence data for SCR and the three DNA sequence datasets for WCR were assembled separately, resulting in 33,593 contigs for SCR, and 3.8 to 4.3 million contigs for WCR (≥200 bp; Table 2). Contigs of at least 200 bp for SCR and 1000 bp for WCR were selected and annotated by BLASTx to search for sequences potentially derived from DNA viruses. BLASTx results showed that multiple contigs from all four samples (ranging from 20 to 26) had “hits” to known nudivirus proteins genes (Supplementary Tables S1 and S2). The 20 contigs from SCR putatively encoding proteins homologous to those from known nudiviruses were further joined into five nudiviral fragments (F1 to F5) totaling 147,179 bp, which was close to a size typical of known nudiviral genomes (Table 1). The sequence read coverage across the five SCR nudiviral genome fragments ranged from 16.1- to 19.5-fold (mean ~18.5-fold) (Table 3).

BLASTx “hits” were obtained to known nudivirus protein coding regions on contigs independently assembled from WCR data in three accessions SRR1107649 (*n* = 26), SRR1107695 (*n* = 25), and SRR1107698 (*n* = 24). There was a total of 75 putative nudivirus-derived contigs across all assemblies. Comparison of nudivirus-derived contigs from these three assemblies indicated that ~70% of the viral sequences were shared among all three. This result indicates that there is a single nudivirus species shared by all three WCR population samples. Further assembly and depletion of duplicated sequences resulted in a final set of 22 unique fragments, a 3.4-fold reduction from the initial 75 fragments. These unique fragments totaled 48,989 bp, or about one-fifth of the average nudivirus genome size (Table 1). Most of the nudiviral sequences from WCR libraries were assembled into short contigs, suggesting that the samples contained limited nudiviral DNA. Sequence homology from BLASTx query results indicated that both the putative SCR and WCR nudivirus sequences are novel nudiviruses. We tentatively name the viruses Diabrotica undecimpunctata howardi nudivirus (DuhNV) and Diabrotica virgifera virgifera nudivirus (DvvNV).

### 3.2. Sequence Analysis of Diabrotica undecimpunctata Howardi Nudivirus (DuhNV)

**Figure 1:**
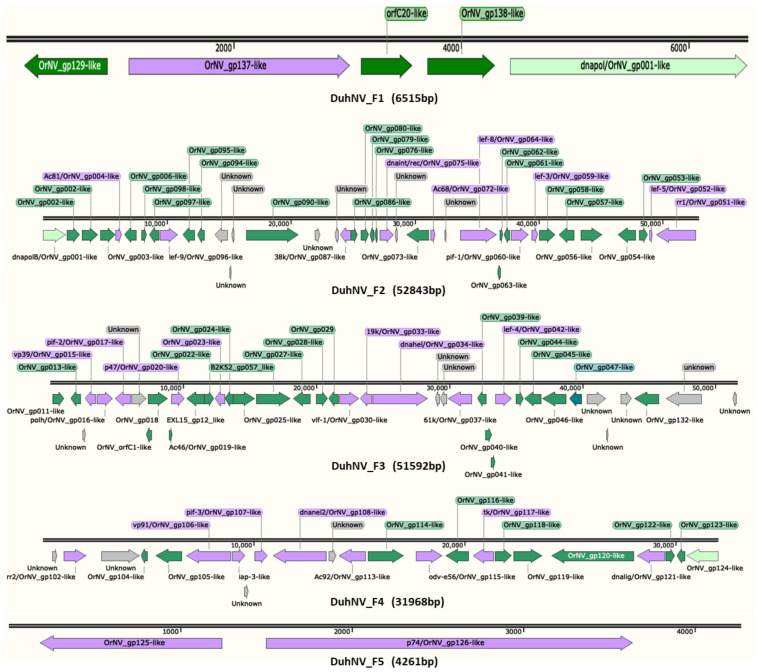
Arrangement of putative genes located on five Diabrotica undecimpunctata howardi nudivirus (DuhNV) genomic fragments (**F1**–**F5**). All ORFs > 50 aa were predicted by BLASTp query against the National Center for Biotechnology Information (NCBI) nr protein database. Arrows indicate ORF direction and orientation. Green, ORFs with BLASTx “hits” to nudiviruses (*E*-values ≤ 0.01); purple, core genes; grey, ORFs for hypothetical genes with unknown protein function; light green, partial ORFs (lacking 5′-start or 3′-stop codons). The maps were generated with SnapGene Viewer v5.2.1.

**Figure 2:**
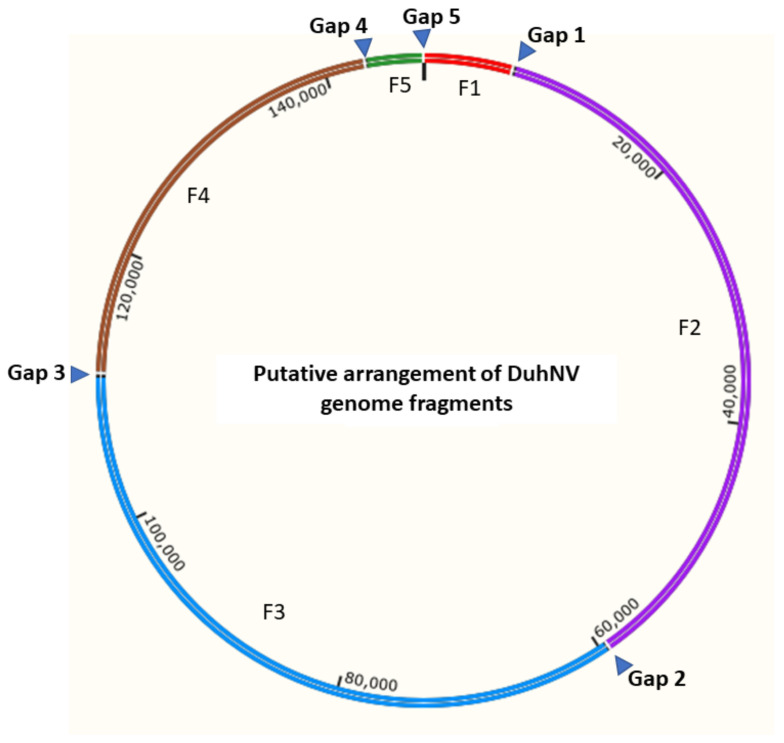
Putative arrangement of fragments **F1–F2–F3–F4–F5** in the 147,179 bp assembled DuhNV genome sequence. Triangles represent sequence gaps. The putative genome schematic was generated by SnapGene Viewer v5.2.1.

The 147,179 bp of the DuhNV genome sequence were assembled from five fragments, with fragment size ranging from 4261 to 52,843 bp (Table 3). The numbers of putative genes encoded by each fragment is also shown in Table 3. The nucleotide composition of DuhNV is about 28.3% G + C, which is similar to the percentage of G + C in the GrBNV genome (28.0%), but much less than that of OrNV (41.6% G + C) (Table 1). The arrangement of the five DuhNV sequence fragments has yet to be confirmed, but available evidence was used to putatively order and orient fragments. Specifically, given that the 5′ end of *dnapol* was located at the 3′ end of DuhNV F1 and the 3′ end of *dnapol* was located at the 5′ end of DuhNV F2, DuhNV F2 was determined to follow DuhNV F1 (Figure 1). The ORFs identified in DuhNV F4 and DuhNV F5 are homologous to the same cluster of genes in OrNV, thus indicating that DuhNV F4 and DuhNV F5 may be adjacent. The two ORFs of DuhNV F5 are homologous to genes OrNV_gp124 and OrNV_gp125, while the 5′ end of DuhNV F1 encodes a gene homologous to OrNV_ gp129 that is positioned downstream of OrNV_gp124 and OrNV_gp125 in the OrNV genome. In the event that the genomic arrangement is conserved between coleopteran nudiviruses, the circular DuhNV genome fragment order was predicted to be F1–F2–F3–F4–F5 (Figure 2).

A total of 109 putative ORFs of > 50 aa were predicted on the five DuhNV genome fragments. The ORFs were named based on the linear order within each fragment (Table 4), and putative structural BLASTp annotations are shown for encoded protein sequences from DuhNV genes and OrNV homologs (Table 4; Supplementary Table S1). Fifteen hypothetical genes of unknown function were predicted in the DuhNV genome (Table 4; Figure 1). The alignment of in silico translated protein sequences encoded by DuhNV genes with nudiviral orthologs indicated that the majority of the DuhNV genes had high sequence identities with the homologous genes of OrNV or nudiviruses isolated from *Drosophila*. The protein sequence identity of 74 predicted DuhNV proteins (~81%) showed >40% sequence identity with those encoded by other nudiviruses (Supplementary Table S1). The protein annotation revealed that the majority of the DuhNV genes are similar to those of known nudiviruses, with 56 of the top BLAST “hits” to homologs from OrNV (Table 4; *E*-values ≤ 6.0 × 10^−8^). The other top “hits” were from nudiviruses isolated from *Drosophila* (Supplementary Table S1), with the exception of DuhNV_F3_ORF35, which showed highest similarity to an ATP-binding cassette (ABC) transporter encoded by Gammaproteobacteria (Supplementary Table S1). Taken together, these results suggest that DuhNV is a novel species within the genus *Alphanudivirus.*

Of the 109 proteins encoded by putative DuhNV ORFs, about 83% had a top BLASTp “hit” to a nudiviral protein represented in the NCBI nr database. These genes included the ~20 core genes of baculoviruses and other essential genes identified in nudiviruses (Table 4). A majority of the DuhNV ORFs show top BLAST “hits” to hypothetical proteins of unknown function previously characterized in other nudivirus genomes. To assess the possible functions, we searched for potential functional domains in the protein sequences encoded by these hypothetical DuhNV genes. CDART results identified known protein domains encoded by 6 of the 24 queried hypothetical DuhNV genes: DvNV_F2_ORF12, _ORF15, DvNV_F3_ORF12, _ORF17, _ORF19, and _ORF35. Most of the unknown DuhNV genes that showed no similarity to previously described nudivirus genes were relatively short ORFs, but some encode relatively large proteins, e.g., DuhNV_F2_ORF12 (329 aa), DuhNV_F3_ORF7 (388 aa), DuhNV_F3_ORF37 (278 aa), DuhNV_F3_ORF39 (868 aa), and DuhNV_F4_ORF3 (616 aa). CDART did not predict any known protein domains encoded by these genes, with the exception of DuhNV_F2_ORF12 that encodes a RING_Ubox (cl7238) zinc-binding domain. RING_Ubox-containing proteins serve various functions, including involvement in virus replication [57], which may reflect the function of this nudivirus ORF.

### 3.3. Analysis of Diabrotica virgifera virgifera Nudivirus Sequences

**Figure 3:**
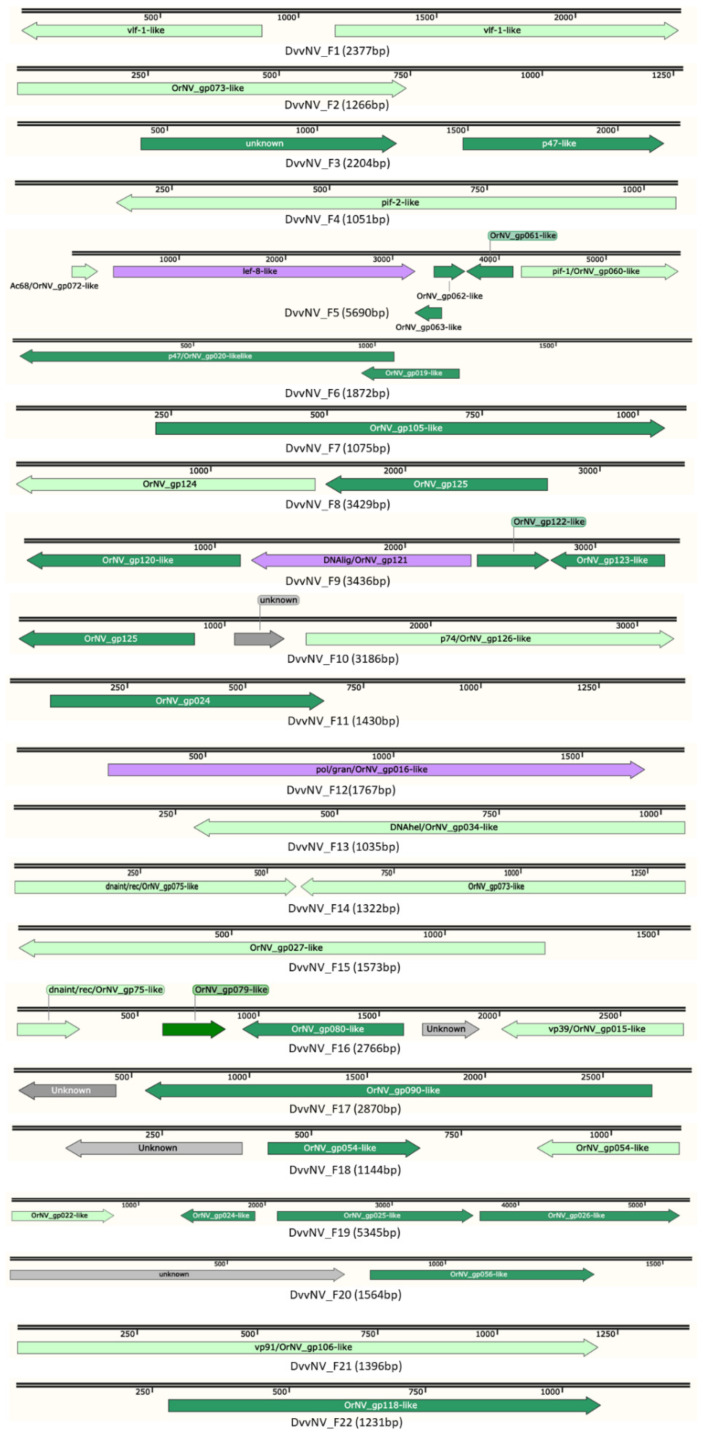
Map of the 22 DvvNV genomic fragments. Arrows indicate ORFs and ORF orientation. Green, ORFs that hit nudiviruses; purple, core genes; grey, unknown ORFs; light green, partial ORFs.

The sequence assembly for the DvvNV genome was not highly contiguous using the available short read data, and resulted in 22 short sequence fragments spanning a total of 53,293 bp (median contig length (N50) = 1867; range 1035 to 5690). Two fragments were >5000 bp in length, and 12 were ≤1872 bp. Despite their relatively short lengths, 48 ORFs were predicted on these 22 DvvNV genome fragments (range 1 to 6 ORFs per contig; Figure 3). Thirteen of the ORFs were considered “partial” sequences due to BLAST alignment coverages of <80%; Supplementary Table S2). Among the 48 predicted DvvNV ORFs, 43 showed similarity to previously described genes from nudiviruses (Table 5), with most top BLAST “hits” to OrNV or nudiviruses isolated from *Drosophila* (*E*-values ≤ 0.009; Supplementary Table S2). Six of the predicted DvvNV ORFs showed no similarity to orthologs from other nudiviruses, and were designated as “unknown” genes. Only 13 core genes were found in the 48 predicted DvvNV ORFs. Pairwise comparisons by BLASTp among the homologous proteins of encoded proteins in the three beetle-infecting nudivirus genomes, DvvNV, DuhNV and OrNV, indicated that most share high (>35%) amino acid sequence identities.

### 3.4. Phylogenetic Analysis of WCR Nudiviruses and Other Nudiviruses

**Figure 4:**
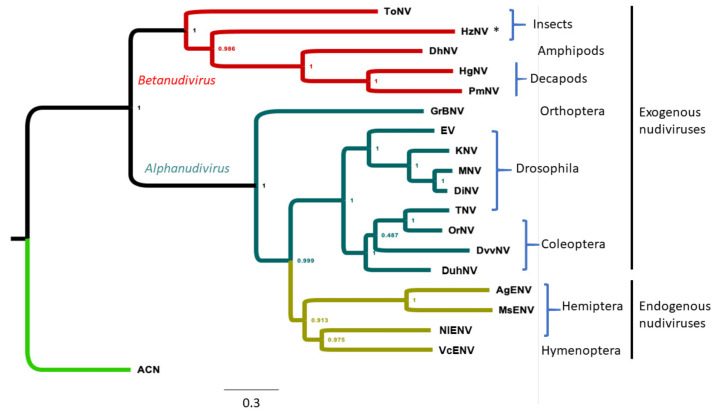
Phylogenetic tree based on nudivirus core sequences. Protein sequences encoded by 31 core genes were downloaded from the NCBI database for 13 sequenced nudiviruses, four endogenous nudiviruses and a baculovirus, *Autographa californica nucleopolyhedrovirus* (ACN, outgroup; Supplementary Table S3). Abbreviations for exogenous nudiviruses from Table 1. AgENV, *Aphis glycines endogenous nudivirus*; MsENV, *Melanaphis sacchari endogenous nudivirus*; NlENV, *Nilaparvata lugens endogenous nudivirus*; VcENV, *Venturia canescens endogenous nudivirus*. Core sequences of AgENV and MsENV were previously reported [26]. The phylogenetic tree was built using supermatrix methods with ETE Toolkit v3. The tree was edited and viewed using FigTree. Taxonomic categorization of host species for most nudiviruses is indicated. * HzNV represents both HzNV1 and HzNV2. Numerals at branch points indicate bootstrap support.

The phylogenetic relationships among DuhNV, DvvNV and other nudiviruses were reconstructed as a supermatrix phylogenetic tree based on aligned protein sequences of core nudiviral genes (Figure 4; Supplementary Table S3). The tree shows two major divisions representing alpha- and beta-nudiviruses. The six betanudiviruses (HzNV represents HzNV1 and HzNV2) were closely related and grouped into a clade distinct from the alphanudiviruses. In the alphanudivirus clade, GrBNV formed an individual branch and was more closely related to ancestral nudiviruses than to other alphanudiviruses. The remaining alphanudiviruses were clustered into two major sub-clades. One sub-clade included a sub-branch of four of the five nudiviruses isolated from *Drosophila*. The exception was TNV, which was more closely related to OrNV and grouped into the sub-branch with the three beetle-infecting nudiviruses. The four endogenous nudiviruses analyzed previously [26] form the second major sub-clade in the alphanudiviral clade.

**Figure 5:**
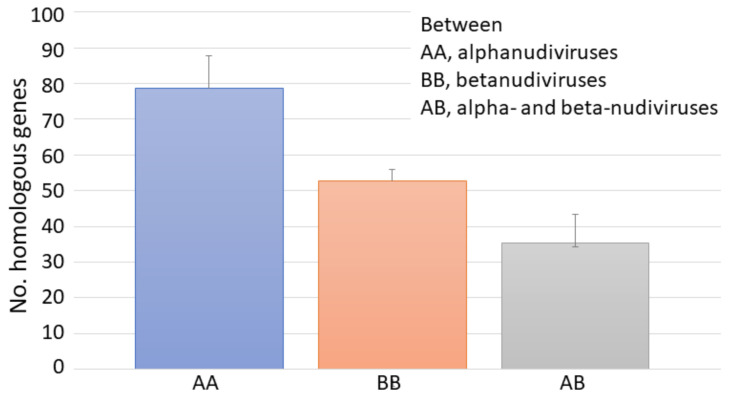
Numbers of homologous proteins among nudiviruses as determined by BLASTp. The cutoff *E*-value for determination of homology was set at 0.0001. Bars represent SD of the mean.

We further analyzed gene similarities within and between the alpha- and beta-nudivirus groups (Figure 5, Supplementary Table S4). Comparison of homologous genes indicated that, on average, ~80 (out of an average nudivirus gene number of ~100) are homologous among *Alphanudivirus*, with ~50 of these *Betanudivirus* genes having high similarities (*E*-values < 0.0001) (Figure 5). Only ~35 genes between alpha- and beta-nudiviruses show high levels of similarity.

## 4. Discussion

Insect-derived DNA and RNA sequence data provide useful resources for metagenomic analyses, including the identification of sequences derived from viruses [42,58]. For this investigation, we report assembled DNA sequences derived from two novel nudiviruses embedded in SCR and WCR short shotgun genomic read data. The 147,179 bp DuhNV genome sequence assembly was considered near complete based on the high representation of genes homologous with those from OrNV, including a core set of protein coding genes. Although near complete, five gaps remain in the DuhNV assembly. Attempts to fill the gaps in the DuhNV genome sequence by PCR were unsuccessful (data not shown). The gaps could contain tandem sequence repeats that make amplification difficult. Third-generation sequencing technologies [59], which provide longer reads, may provide a means for completion of both the DuhNV and DvvNV genome sequences. Putative nudiviral particles were reported from SCR field populations more than three decades ago [16]; however, it remains unknown if those particles were from the same DuhNV species described herein. Given the assembly of the majority of the DuhNV genome (Figure 1; Figure 2) at read coverage depths >18-fold (Table 2), the viral titer was likely high in the Iowa SCR population sample used for this work. Despite the previous observation of nudivirus-like particles [16] and discovery of DuhNV sequences described herein, it is not possible to comment on nudivirus distribution in the U.S. as these insects move very long distances: SCR migrate to the northern states in late spring or early summer from their overwintering range along the Gulf Coast [60,61], in contrast to WCR, which are present in the northern states year-round [62].

DNA viruses have not previously been documented from WCR. The discovery of DvvNV-derived sequences within short shotgun genomic read data from WCR suggests that a nudivirus infects WCR. The viral sequences were observed in WCR populations from three Midwestern states, suggesting the virus has a wide geographic distribution. However, due to relatively low read coverage, the DvvNV genome sequence assembly was highly fragmented, preventing detailed characterization. The low level of viral sequences in these WCR genomic DNA samples may be due to a low rate of DvvNV infection. The sample size of five used for WCR DNA isolation and sequencing [42] compared to 71 SCR adults pooled from the Iowa sample suggests that sampling bias may have impacted our results. Low recovery also may be due to lower viral titers in each of the WCR individuals that were sampled. A third possible explanation for the presence of limited number of DvvNV sequences is that the DvvNV-derived reads are from an endogenous nudivirus. The insertion of nudivirus genomic sequence into the host genome can involve loss or disruption of most of the sequence. To investigate this possibility, we used the same methods described herein to examine DNA sequence data and assembled sequences from more than five datasets for WCR collected from disparate geographical locations in the U.S. (Arizona, Pennsylvania, Iowa and South Dakota), and from Hungary. No nudivirus sequences were identified in these additional datasets when queried with homologous nudiviruses sequences using the BLASTn. Hence, DvvNV is unlikely to be an endogenous nudivirus as DvvNV sequence would otherwise have been present in the genomes of other WCR. In addition, phylogenetic analysis of nudiviruses grouped DvvNV into the exogenous nudivirus clade, rather than the clade composed of endogenous nudiviruses. Hence, we conclude that DvvNV is likely an exogenous alphanudivirus (Figure 4).

The supermatrix phylogenetic tree based on 31 core nudiviral genes showed an interesting evolutionary relationship among 18 nudiviruses, including DuhNV and DvvNV described here. This phylogeny suggests that alpha- and beta-nudiviruses evolved independently from ancestral dsDNA viruses. The bifurcation of marsh crane fly-infecting nudivirus ToNV and HzNV, from the moth pest *Helicoverpa* (formerly *Heliothis*) *zea*, is the closest to the base of the phylogeny rooted by the ACN outgroup. Analysis of the similarity of homologous genes between alpha- and betanudiviruses (Figure 5) suggests that the genes of the betanudiviruses, for which we have sequences, are more diverse than those of alphanudiviruses. This suggests a greater evolutionary time over which mutations could have accumulated, and is consistent with the phylogenetic analysis based on nudivirus core genes (Figure 4). Based on the nudivirus sequences available to date, however, we can only conclude that the alpha- and betanudiviruses are derived from a common ancestor and that the two groups are, therefore, of the same evolutionary age.

**Figure 6:**
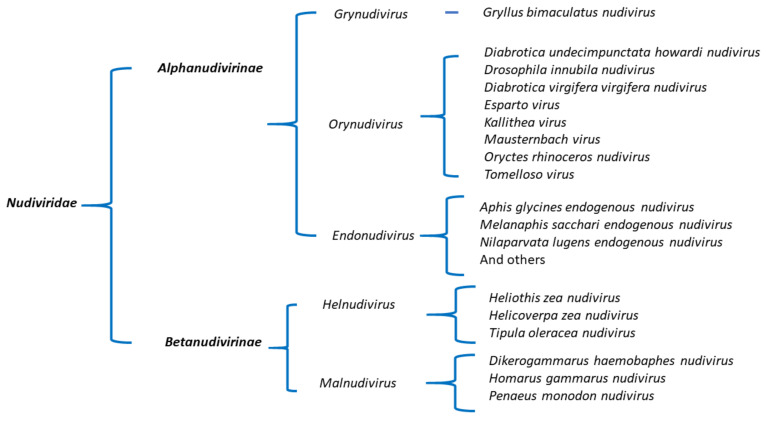
Proposed new taxonomic classification of *Nudiviridae.* Two subfamilies and five genera are proposed.

The phylogenetic analysis clearly divided nudiviruses into two clusters, namely alpha- and beta- nudiviruses. However, the phylogenetic tree also suggests that the current taxonomic classification of *Nudiviridae* can be further expanded. Based on our analysis, we propose that alpha- and beta- nudiviruses could be assigned to two subfamilies, *Alphanudivirinae* and *Betanudivirinae*, under *Nudiviridae* (Figure 6). In *Alphanudivirinae*, GrBNV is in a separate clade, distant from other alphanudiviral species (Figure 4). GrBNV shared only 63–72 homologous genes with other alphanudiviruses (Supplementary Table S3), while the other nudiviruses in this subfamily share similar numbers (75–90) of homologous genes and were isolated from *Drosophila* or beetles. We propose that GrBNV represents a genus, *Grynudivirus* (Gry from *Gryllus*), and OrNV represents the type species of the other genus, named *Orynudivirus*. Notably, endogenous nudiviruses, e.g., *Aphis glycines endogenous nudivirus,* are in the branch of the *Alaphanudivirinae*, and form a unique clade. We propose the name of *Endonudivirus* for the genus of endogenous nudiviruses that are integrated in host genomes. Hence *Alphanudivirinae* contains three new genera. Similarly, two novel genera could be established in the *Betanudivirinae* based on the class of host species: *Helnudivirus* and *Malnudivirus*. The name *Helnudivirus* is from HzNV, which includes three nudiviral species isolated from the class Insecta. The three nudiviruses isolated from the class *Malacostraca* could be assigned a new genus, *Malnudivirus*. The proposed new nudiviral classification, based on our phylogenetic tree (Figure 4), and evidence of homologous gene similarities among the nudiviruses (Figure 5), is illustrated in Figure 6.

The discovery of sequences derived from two novel nudiviruses from corn rootworms increases our understanding of diversity within the rootworm virome, and sheds light on the evolution of this remarkable virus family. Analysis of virus-derived sequences from a limited number of SCR, WCR and NCR (unpublished data) DNA sequence datasets indicated that DuhNV sequences were observed only from SCR, and that DvvNV sequences were found only in WCR. A large-scale sequence investigation, in addition to experimentation, would be required to determine the host range of each of these viruses. Further work is needed to complete the genome sequences of DuhNV and DvvNV, to examine the distribution and host range of these viruses, and to address their impact on corn rootworm populations. Furthermore, our analyses suggest that revision of the current taxonomic classification of *Nudiviridae* may be warranted.

## Figures and Tables

**Table 1:** Characteristics of exogenous nudivirus genome sequences (current as of December 2020). NA, not applicable.

Virus	Abbr.	Length (bp)	Accession	No. ORFs	%G + C	Host	Isolates	Ref
***Alphanudivirus***								
Gryllus bimaculatus nudivirus	GrBNV	96,944	NC_009240.1	98	27.99	*Gryllus bimaculatus*	Starnberg, Germany	[28,29]
Kallithea virus	KNV	152,388	NC_033829.1	95	32.88	*Drosophila melanogaster*	Kharkiv, Ukraine	[30]
Drosophila innubila nudivirus	DiNV	155,555	NC_040699.1	106	30.29	*Drosophila innubila*	Arizona, USA	[31]
Oryctes rhinoceros nudivirus	OrNV	127,615	EU747721.1	139	41.63	*Oryctes rhinoceros*	Malaysia	[32,33]
Esparto virus	EV	183,261	NC_040536.1	87	29.45	*Drosophila melanogaster*	Esparto, CA, USA	NA
Mauternbach virus	MNV	154,465	MG969167.1	93	30.67	*Drosophila melanogaster*	DrosEU50 Mauternbach 2015, Austria	NA
Tomelloso virus	TNV	112,307	NC_040789.1	93	39.63	*Drosophila melanogaster*	DrosEU28 Tomelloso 2015, Spain	NA
***Betanudivirus***								
Heliothis zea nudivirus 1	HzNV1	228,089	AF451898.1	154	41.81	*Heliothis zea*	Taiwan, PRC	[34,35]
Helicoverpa zea nudivirus 2	HzNV2	231,621	NC_004156.2	113	41.88	*Helicoverpa zea*	Massachusetts, USA	[36,37]
Homarus gammarus nudivirus	HgNV	107,063	MK439999.1	97	35.03	*Homarus gammarus*	52S104HLG2, hepatopancreas, UK	[38]
Penaeus monodon nudivirus	PmNV	119,638	NC_024692.1	115	34.53	*Penaeus monodon*	Indonesia	[39]
Tipula oleracea nudivirus	ToNV	145,704	NC_026242.1	131	25.53	*Tipula oleracea*	England, UK	[40]
Dikerogammarus haemobaphes nudivirus	DhNV	119,754	MT488302	106		*Dikerogammarus haemobaphes*	UK	[41]
**Unclassified**								
Allomyrina virus		NA	AIY68660	NA	NA	*Allomyrina dichotoma*	Youngdong-1, Korea	NA

**Table 2:** Summary of southern corn rootworm (SCR) and western corn rootworm (WCR) sequence assembly and identification of nudivirus-derived sequences.

Species	Sequencing Dataset	U.S. Collection Site	Total Reads (M)	Trimmed Reads (M)	Average Quality Score (*q*)	N50 (bp)	No. of Contigs (≥200)	No. of Contigs (≥1000)
*D. undecimpunctata*	SRR13364002	Ames, IA	9.7	9.7	37	420	33,539	NA
*D. virgifera virgifera*	SRR1107649	Adams County, IN	195	184	37	403	4.4 M	84,419
*D. virgifera virgifera*	SRR1107695	Seneca, KS	194	186	38	414	4.1 M	71,902
*D. virgifera virgifera*	SRR1107698	Colfax, NE	41	40	37	422	3.8 M	97,060

**Table 3:** Sequence read coverage of five nudivirus genomic fragments (F1 to F5) from *D. undecimpunctata*.

Fragment	Length (bp)	G + C (%)	No. Putative CDS	Total Reads Mapped	Total Bases Mapped	Read Coverage
F1	6515	28.49	5	1210	121,000	18.6
F2	52,843	27.93	40	10,280	1,028,000	19.5
F3	51,592	27.88	40	9858	985,800	19.1
F4	31,968	27.56	23	6202	620,200	19.4
F5	4261	29.46	2	687	68,700	16.1
Total	147,179		109	28,237	2,823,700	
Average		28.264				18.5

**Table 4:** Putative genes of Diabrotica undecimpunctata howardi nudivirus (DuhNV).

ORF	Gene/Domain	Length (aa)	OrNV Homologs
**Replication, Repair and Recombination of DNA**			
DuhNV_F1_ORF5 *	***Dnapol***	695	OrNV_gp001
DuhNV_F2_ORF1 *	***Dnapol***	633	OrNV_gp001
DuhNV_F3_ORF23	***Dnahel***	1390	OrNV_gp034
DuhNV_F2_ORF23	*dnaint/rec*	389	OrNV_gp075
DuhNV_F2_ORF33	***lef-3***	177	OrNV_gp059
DuhNV_F4_ORF10	*dnahel2*	839	OrNV_gp108
DuhNV_F4_ORF20	*Dnalig*	423	OrNV_gp121
**Nucleotide Metabolism**			
DuhNV_F2_ORF40	*rr1*	1045	OrNV_gp051
DuhNV_F4_ORF2	*rr2*	358	OrNV_gp102
DuhNV_F1_ORF2	*tk*/GrBNV_gp17-like	648	OrNV_gp137
DuhNV_F3_ORF31	*tk/GrBNV-gp74_like*	197	OrNV_gp044
DuhNV_F4_ORF16	*tk(guanosine monophosphate kinase)*	321	OrNV_gp117
DuhNV_F5_ORF1	*tk/GrBNV_gp044_like*	352	OrNV_gp125
**Transcription**			
DuhNV_F3_ORF11	***p47***	324	OrNV_gp020
DuhNV_F3_ORF30	***lef-4***	417	OrNV_gp042
DuhNV_F2_ORF39	***lef-5***	82	OrNV_gp052
DuhNV_F2_ORF28	***lef-8***	988	OrNV_gp064
DuhNV_F2_ORF9	***lef-9***	505	OrNV_gp096
DuhNV_F3_ORF21	***vlf-1***	504	OrNV_gp030
**Oral Infectivity**			
DuhNV_F5_ORF2	***p74***	712	OrNV_gp126
DuhNV_F2_ORF32	***pif-1***	489	OrNV_gp060
DuhNV_F3_ORF6	***pif2***	376	OrNV_gp017
DuhNV_F4_ORF9	***pif-3***	202	OrNV_gp107
**Packaging, Assembly, and Morphogenesis**			
DuhNV_F3_ORF5	*polh/gran*	404	OrNV_gp016
DuhNV_F3_ORF22	***19k/pif-5***	281	OrNV_gp033
DuhNV_F2_ORF26	***Ac68***	136	OrNV_gp072
DuhNV_F2_ORF18	***38k***	276	OrNV_gp087
**Inhibition of Apoptosis**			
DuhNV_F4_ORF7	***iap-3***	202	OrNV_gp134
**Unknown Function**			
DuhNV_F2_ORF5	***Ac81***	173	OrNV_gp004
DuhNV_F3_ORF4	***vp39***	250	OrNV_gp015
DuhNV_F4_ORF6	***vp91***	695	OrNV_gp106
DuhNV_F4_ORF12	***Ac92***	410	OrNV_gp113
DuhNV_F4_ORF14	***odv-e56***	420	OrNV_gp115
**Other**			
DuhNV_F1_ORF1	ND	240	OrNV_gp129/gp136
DuhNV_F1_ORF3	ND	149	OrNV_orfC20
DuhNV_F1_ORF4	ND	594	OrNV_gp138
DuhNV_F2_ORF2	trypsin-like serine protease-like protein	339	OrNV_gp002
DuhNV_F2_ORF3	trypsin-like serine protease-like protein	427	OrNV_gp002
DuhNV_F2_ORF4	ND	395	OrNV_gp003
DuhNV_F2_ORF6	thymidylate synthase/pyrimidine hydroxymethylase-like	296	OrNV_gp006
DuhNV_F2_ORF7	ND	143	OrNV_gp098
DuhNV_F2_ORF8	mRNA decapping enzyme 2-like	245	OrNV_gp097
DuhNV_F2_ORF10	ND	314	OrNV_gp095
DuhNV_F2_ORF11	ND	162	OrNV_gp094
DuhNV_F2_ORF12	cl17238, RING-Ubox	329	no hit
DuhNV_F2_ORF13	None	62	no hit
DuhNV_F2_ORF14	None	63	no hit
DuhNV_F2_ORF15 ^1^	cl25745, pfam05764	1412	OrNV_gp090
DuhNV_F2_ORF16	None	148	no hit
DuhNV_F2_ORF17	None	78	no hit
DuhNV_F2_ORF19	ND	189	OrNV_gp086
DuhNV_F2_ORF20	ND	227	OrNV_gp080
DuhNV_F2_ORF21	ND	89	OrNV_gp079
DuhNV_F2_ORF22	ND	52	OrNV_gp076
DuhNV_F2_ORF24	None	61	no hit
DuhNV_F2_ORF25	ND	593	OrNV_gp073
DuhNV_F2_ORF27	None	56	no hit
DuhNV_F2_ORF29	ND	74	OrNV_gp063
DuhNV_F2_ORF30	ND	88	OrNV_gp062
DuhNV_F2_ORF31	ND	140	OrNV_gp061
DuhNV_F2_ORF34	HZV_115-like protein	425	OrNV_gp058
DuhNV_F2_ORF35	patatin-like phospholipase-like protein	397	OrNV_gp057
DuhNV_F2_ORF36	ND	576	OrNV_gp056
DuhNV_F2_ORF37	ND	458	OrNV_gp054
DuhNV_F2_ORF38	ND	234	OrNV_gp053
DuhNV_F3_ORF1	mitochondrial carrier protein-like protein	293	OrNV_gp011
DuhNV_F3_ORF2	ND	231	OrNV_gp013
DuhNV_F3_ORF3	None	76	no hit
DuhNV_F3_ORF7	None	388	no hit
DuhNV_F3_ORF8	ND	131	OrNV_orfC1
DuhNV_F3_ORF9	ND	509	OrNV_gp018
DuhNV_F3_ORF10	Ac46	89	OrNV_gp019
DuhNV_F3_ORF12 ^2^	EXL15_gp12_like/DUF4679 (pfam15728)	432	no hit
DuhNV_F3_ORF13	ND	241	OrNV_gp022
DuhNV_F3_ORF14	guanylate kinase-like protein	245	OrNV_gp023
DuhNV_F3_ORF15	ND	196	OrNV_gp024
DuhNV_F3_ORF16	ND	550	OrNV_gp025
DuhNV_F3_ORF17 ^3^	B2K52_gp057/Smc cl341474	858	no hit
DuhNV_F3_ORF18	ND	422	OrNV_gp027
DuhNV_F3_ORF19 ^4^	PPK13561/cl32896, EAL domain containing protein	297	OrNV_gp028
DuhNV_F3_ORF20	ND	232	OrNV_gp029
DuhNV_F3_ORF24	None	96	no hit
DuhNV_F3_ORF25	None	157	no hit
DuhNV_F3_ORF26	61k AcORF9	575	OrNV_gp037
DuhNV_F3_ORF27	ND	199	OrNV_gp039
DuhNV_F3_ORF28	ND	166	OrNV_gp040
DuhNV_F3_ORF29	ND	110	OrNV_gp041
DuhNV_F3_ORF32	ND	403	OrNV_gp045
DuhNV_F3_ORF33	ND	591	OrNV_gp046
DuhNV_F3_ORF34	ND	293	OrNV_gp047
DuhNV_F3_ORF35 ^5^	cl34121, energy-coupling factor ABC transporter	490	no hit
DuhNV_F3_ORF36	None	66	no hit
DuhNV_F3_ORF37	None	278	no hit
DuhNV_F3_ORF38	ND	592	OrNV_gp132
DuhNV_F3_ORF39	None	868	no hit
DuhNV_F3_ORF40	None	85	no hit
DuhNV_F4_ORF1	None	76	no hit
DuhNV_F4_ORF3	None	616	no hit
DuhNV_F4_ORF4	ND	91	OrNV_gp104
DuhNV_F4_ORF5	ND	408	OrNV_gp105
DuhNV_F4_ORF8	None	68	no hit
DuhNV_F4_ORF11	None	120	no hit
DuhNV_F4_ORF13	ND	566	OrNV_gp114
DuhNV_F4_ORF15	ND	345	OrNV_gp116
DuhNV_F4_ORF17	ND	266	OrNV_gp118
DuhNV_F4_ORF18	ND	454	OrNV_gp119
DuhNV_F4_ORF19	ND	1285	OrNV_gp120
DuhNV_F4_ORF21	ND	143	OrNV_gp122
DuhNV_F4_ORF22	ND	124	OrNV_gp123
DuhNV_F4_ORF23	ND	476 *	OrNV_gp124

Bold, baculovirus core genes; italic, common nudivirus genes. *, separate fragments of the same gene; ND, domain search not done for genes with *Oryctes rhinoceros nudivirus* (OrNV) homologs; none, no pfam domains identified. ^1^ YL1 nuclear protein; ^2^ cl29686, domain of unknown function (DUF4679); ^3^ chromosome segregation ATPase (cell cycle control, cell division, chromosome partitioning); ^4^ putative diguanylate cyclase (provisional); ^5^ ABC transporter protein.

**Table 5:** Sequence analysis of Diabrotica virgifera virgifera nudivirus (DvvNV). Baculovirus core genes are shown in bold. Genes common to nudiviruses are shown in italics.

ORF	Length (Some Partial)	Gene	Similar OrNV ORF	Similar DuhNV ORF
DvvNV_F1_ORF1	414	***vlf-1***	OrNV_gp030	DuhNV_F3_ORF21
DvvNV_F1_ORF2	289	***vlf-1***	OrNV_gp030	DuhNV_F3_ORF21
DvvNV_F2_ORF1	246	OrNV_gp073-like	OrNV_gp073	DuhNV_F2_ORF25
DvvNV_F3_ORF1	223	***p47***	OrNV_gp020	DuhNV_F3_ORF11
DvvNV_F3_ORF2	284	no hit		DuhNV_F3_ORF9
DvvNV_F4_ORF1	293	***pif-2***	OrNV_gp017	DuhNV_F3_ORF6
DvvNV_F5_ORF1	82	Ac68-like	OrNV_gp072	no hit
DvvNV_F5_ORF2	943	***lef-8***	OrNV_gp064	DuhNV_F2_ORF28
DvvNV_F5_ORF3	81	OrNV_gp063-like	OrNV_gp063	no hit
DvvNV_F5_ORF4	98	OrNV_gp062-like	OrNV_gp062	DuhNV_F2_ORF30
DvvNV_F5_ORF5	144	GrBNV_gp51-like	OrNV_gp061	no hit
DvvNV_F5_ORF6	493	***pif-1***	OrNV_gp060	DuhNV_F2_ORF32
DvvNV_F6_ORF1	343	***p47***	OrNV_gp020	DuhNV_F3_ORF11
DvvNV_F6_ORF2	89	Ac146-like	OrNV_gp019	DuhNV_F3_ORF10
DvvNV_F7_ORF1	272	GrBNV gp43-like	OrNV_gp105	DuhNV_F4_ORF5
DvvNV_F8_ORF1	379	GrBNV_gp44-like	OrNV_gp125	DuhNV_F5_ORF1
DvvNV_F8_ORF2	512	OrNV_gp124-like	OrNV_gp124	DuhNV_F4_ORF23
DvvNV_F9_ORF1	373	GrBNVgp37-like	OrNV_gp120	DuhNV_F4_ORF19
DvvNV_F9_ORF2	384	***DNAlig***	OrNV_gp121	DuhNV_F4_ORF20
DvvNV_F9_ORF3	125	GrBNV_gp39-like	OrNV_gp122	no hit
DvvNV_F9_ORF4	199	GrBNVgp41-like	OrNV_gp123	DuhNV_F4_ORF22
DvvNV_F10_ORF1	284	GrBNV_gp44-like	OrNV_gp125	DuhNV_F5_ORF1
DvvNV_F10_ORF2	80	unknown		no hit
DvvNV_F10_ORF3	596	***p74***	OrNV_gp126	DuhNV_F5_ORF2
DvvNV_F11_ORF1	193	GrBNVgp75-like	OrNV_gp024	DuhNV_F3_ORF15
DvvNV_F12_ORF1	474	*pol/gran*	OrNV_gp016	DuhNV_F3_ORF5
DvvNV_F13_ORF1	251	***DNAhel***	_OrNV_gp034	DuhNV_F3_ORF23
DvvNV_F14_ORF1	184	*dnaint/rec*	OrNV_gp075	DuhNV_F2_ORF23
DvvNV_F14_ORF2	251	putative gene	OrNV_gp073	DuhNV_F2_ORF25
DvvNV_F15_ORF1	411	GrBNV_gp78-like	OrNV_gp027	DuhNV_F3_ORF18
DvvNV_F16_ORF1	86	*dnaint/rec*	OrNV_gp075	DuhNV_F2_ORF23
DvvNV_F16_ORF2	222	GrBNVgp60-like	OrNV_gp080	no hit
DvvNV_F16_ORF3	86	GrBNVgp59-like	OrNV_gp079	DuhNV_F2_ORF21
DvvNV_F16_ORF4	79	unknown		
DvvNV_F16_ORF5	250	vp39	OrNV_gp015	DuhNV_F3_ORF4
DvvNV_F17_ORF1	715	GrBNVgp28-like	OrNV_gp090	DuhNV_F2_ORF15
DvvNV_F17_ORF2	136	unknown		
DvvNV_F18_ORF1	84	GrBNV gp83-like	OrNV_gp054	DuhNV_F2_ORF37
DvvNV_F18_ORF2	97	unknown		DuhNV_F2_ORF37
DvvNV_F18_ORF3	78	GrBNV gp83-like	OrNV_gp054	no hit
DvvNV_F19_ORF1	270	GrBNV_gp72-like	OrNV_gp022	DuhNV_F3_ORF13
DvvNV_F19_ORF2	516	GrBNV_gp76-like	OrNV_gp025	DuhNV_F3_ORF16
DvvNV_F19_ORF3	526	unknown	OrNV_gp026	DuhNV_F3_ORF17
DvvNV_F19_ORF4	193	GrBNVgp75-like	OrNV_gp024	DuhNV_F3_ORF15
DvvNV_F20_ORF1	171	OrNV_gp056	OrNV_gp056	DuhNV_F2_ORF36
DvvNV_F20_ORF2	256	unknown		
DvvNV_F21_ORF1	402	***vp91***	OrNV_gp106	DuhNV_F4_ORF6
DvvNV_F22_ORF1	263	GrBNV_gp35-like	OrNV_gp118	DuhNV_F4_ORF17

## Data Availability

Raw sequence data used for this analysis are available as indicated in Section 2.1 of the manuscript. The sequences of DuhNV and DvvNV are provided as text files in Supplementary Materials. GenBank accession numbers DuhNV_F1 to 5, MW503925 to 9 respectively.

## Supplementary Materials

The following Supplementary Materials are available online at https://www.mdpi.com/1999-4915/13/2/269/s1. Table S1, Analysis of DuhNV ORFs; Table S2, Analysis of DvvNV ORFs; Table S3, List of core genes used for phylogenetic analysis; Table S4, Number of homologous genes among nudiviruses. Virus sequence text files: DuhNV_sequence, DvvNV_gb_format.

## Author Notes

A 2020 ICTV proposal that places HgNV and PmNV into a new genus *Gammanudivirus*, and ToNV into a new genus *Deltanudivirus*, has come to our attention: https://talk.ictvonline.org/files/proposals/animal_dna_viruses_and_retroviruses/m/animal_dna_ec_approved/10386 These changes will be added to the 2020 release of official virus taxonomy (https://talk.ictvonline.org/taxonomy/) after ratification by the ICTV in 2021. While the supermatrix tree presented in the current manuscript (Figure 4) does not support creation of additional nudivirus genera, the reader is referred to the ICTV proposal to review additional analyses that provide the basis for the revised classification.
